# Phytochemicals in Chronic Disease Prevention

**DOI:** 10.3390/nu15234933

**Published:** 2023-11-27

**Authors:** Jing Sun, Shuwei Luo, Jianjun Deng, Haixia Yang

**Affiliations:** 1 College of Food Science and Nutritional Engineering, China Agricultural University, Beijing 100083, China; 2 State Key Laboratory of Vegetable Biobreeding, Institute of Vegetables and Flowers, Chinese Academy of Agricultural Sciences, Beijing 100081, China; dengjianjun@nwu.edu.cn

Chronic diseases, also known as noncommunicable diseases (NCD), are characterized by long durations and a slow progression of the associated medical conditions. According to the World Health Organization, they are responsible for 74% of annual deaths worldwide and have become a global pandemic with high incidence rates [1]. The development of chronic diseases is influenced by several factors, including physical activity, dietary patterns, tobacco and alcohol consumption, and environmental factors. For example, refined sugar intake and ultra-processed food products are now recognized as the most important risk factors for the development of NCD. Consequently, interventions intended to not only treat but also prevent the progression of chronic diseases early in life have become crucial [2,3]. Importantly, diet plays a vital role in promoting health and preventing chronic diseases. Phytochemicals, such as polyphenols, terpenoids, carotenoids, and alkaloids, are relatively small molecular compounds isolated from edible plants that exhibit various pharmacological activities in relation to chronic ailments such as obesity, diabetes, and cardiovascular diseases [4,5]. Therefore, the investigation of the relationship between phytochemicals and chronic disease prevention has emerged as an important topic [6,7]. This Special Issue, “Phytochemicals and Prevention of Chronic Diseases”, features a series of high-quality research articles that explore the isolation, identification, and bioactivities of phytochemicals, as well as the underlying molecular mechanisms involved in chronic diseases through antioxidation, neuroprotection, and the modulation of gut microbiota.

Clinical trials have shown that natural phytochemicals can improve blood glucose levels and lipid accumulation. Lycopene, a lipophilic unsaturated carotenoid, has been found to significantly restore blood glucose levels in 152 type 2 diabetes patients based on publications from the past 5 years. This effect is attributed to the accumulation of leptin in plasma, a reduction in oxidized low-density lipoprotein cholesterol, and the amelioration of oxidative stress [8]. Furthermore, flaxseed, soy, and red clover, rich in proteins and isoflavones, have been shown to improve serum lipids by inhibiting total cholesterol and levels of low- and high-density lipoprotein cholesterol. This beneficial effect has demonstrated potential in preventing cardiovascular diseases in postmenopausal women [9]. Moreover, an increasing trend in childhood obesity has been observed worldwide, highlighting the need for the regulation of dietary food intake. Statistical analysis based on Korean preschoolers (aged 3–5 years) revealed an inverse correlation between the quartiles of dietary phytochemical intake and overweight/obesity, particularly among boys who consume high amounts of meat, milk, and dairy products [10]. Overall, these findings emphasize the importance of natural phytochemicals in improving blood glucose levels, lipid profiles, and obesity management, specifically in relation to preventing cardiovascular diseases and promoting healthy dietary habits. Meanwhile, public health policymakers should pay attention to these results so that further policies and strategies for the management of overweight/obesity can be proposed. 

Meanwhile, the consumption of phytochemicals, such as ginsenoside Rh4, zerumbone, rosmarinic acid, and schisandrin B, has been widely recognized for its potential in preventing and treating chronic diseases in animal models. Ginsenoside Rh4, a terpenoid compound derived from ginseng, is composed of triterpene aglycones and glycosides. In a mouse model of esophageal squamous cell carcinoma, promising inhibitory effects on cancer cell metastasis were induced by Ginsenoside Rh4 administration through the regulation of the Wnt/β-catenin pathway. Additionally, Ginsenoside Rh4 demonstrated the ability to inhibit the migration and invasion of gastric cancer cells in vivo by reversing epithelial–mesenchymal transition induced by the suppression of the TGF-β/Smad2/3 signaling axis [11,12]. Furthermore, another phytochemical compound, sesquiterpenoid zerumbone, exerted therapeutic effects on adult mouse microglia by reducing the expression of lipocalin-2 as well as pro-inflammatory cytokines (interleukin-6 and -1β and tumor necrosis factor-α) and chemokines (CCL-2 and CXCL-10). Additionally, zerumbone facilitated the polarization of macrophages into M2 phenotypes and regulated cellular redox homeostasis by activating the AMPK and Akt/GSK3β signaling pathways in vitro [13]. Regarding rosmarinic acid, it was reported to exhibit neuroprotective properties against rotenone-induced human neuroblastoma SH-SY5Y cell injury via restoring mitochondrial function. This effect was achieved through upregulating the expression of peroxisome proliferator-activated receptor gamma coactivator 1 and the phosphorylation of Akt and AMPK while suppressing the hyperphosphorylation of Abl [14]. Moreover, schisandrin B, a dibenzooctadiene lignan derived from *Schisandra chinensis*, exhibited a protective effect in a mouse model of ethanol-induced liver and brain injury. It achieved this by inhibiting inflammasome activation and collagen deposition and preventing neurological defects as supported by neurological tests. Surprisingly, schisandrin B showed a therapeutic effect rather than a preventive one in ethanol-induced liver injury [15]. Additionally, a molecular docking analysis revealed strong binding ability between 11-O-(4′-O-methylgalloyl)-bergenin, a potential anti-inflammatory compound isolated from *Saxifraga atrata*, and arachidonic acid 15-lipoxygenase, nitric oxide synthase, epidermal growth factor receptor 2, and e-selectin. This compound may exert its effects through the MAPK and NF-κB signaling pathways [16]. Overall, these findings highlight the potential of phytochemicals, such as ginsenoside Rh4, zerumbone, rosmarinic acid, schisandrin B, and 11-O-(4′-O-methylgalloyl)-bergenin, in the prevention and treatment of chronic diseases. Further studies are warranted to explore their mechanisms of action and potential clinical applications.

The exploration of bioactivities for phytochemicals has been limited due to the low extraction rate of pure phytochemicals. Multiple studies have utilized raw plant extracts to investigate the functions of these phytochemicals. The ethanol extract obtained from *Mitragyna speciosa (Korth.) Havil.* Leaves, which includes phenolic compounds, flavonoids, and alkaloids, exhibited significant inhibition of α-glucosidase and pancreatic lipase activities. Mitragynine, identified as the main alkaloid in the ethanol extract, noncompetitively inhibited α-glucosidase activity and synergistically interacted with acarbose, suggesting its potential in preventing diabetes mellitus [17]. Furthermore, the ethyl acetate-n-butanol extracts of *Allium tenuissimum* L. flowers, abundant in flavonoid content (1429.5 μg/g extract), particularly kaempferol (284.1 μg/g flavonoids), demonstrated strong α-glucosidase inhibition activities in vitro. In addition, these extracts ameliorated glycolipid metabolic disorders and inflammation in diabetic mice [18]. However, green extraction strategies, such as water/hydro extraction methods, should be encouraged instead of the use of organic solvents due to the side effects and toxicity of extracts as well as environmental pollution. A mixture of aqueous extracts derived from red grape, blackcurrant, redcurrant, rosehip, and black cherry was characterized by the presence of 1.74 mg/L trans-resveratrol and 2.37 mg/L trans-piceid. This mixture exhibited anticancer effects by inhibiting the expression levels of DNA methyltransferases (DNMT1, DNMT3A, and DNMT3B) and histone deacetylases (HDAC2 and HDAC3) in a mouse model exposed to the carcinogen 7,12-dimethylbenz(a)anthracene [19]. Recently, fruit juice consumption has become more popular than the consumption of fresh fruits. Notably, not-from-concentrate apple juice with minimal processing showed higher total phenol content (2169.03 ± 116.84 GAE mg/100 mL) compared to from-concentrate apple juice (1073.32 ± 72.33 GAE mg/100 mL). In a healthy SD rat model, the former significantly alleviated intestinal inflammation and maintained intestinal homeostasis in the gut [20].

The exploration and purification methods of compounds in plants play a crucial role in studying the bioactivities of phytochemicals. In this regard, affinity ultrafiltration-HPLC, a high-throughput screening technique, has been employed to identify potential active ingredients with anti-inflammatory properties that can interact with cyclooxygenase-2 (COX-2), an inflammation-related isoenzyme, in the methanol/water extracts of *Saxifraga atrata* [16]. Through the application of this method, the phenolic compound 11-O-(4′-O-methylgalloyl)-bergenin was rapidly identified as the target component (purity > 99%) and demonstrated significant anti-inflammatory activities exerted via the AA metabolism, MAPK, and NF-κB signaling pathways [16]. The further development of more efficient strategies is required to deepen our understanding of the bioactivities exhibited by phytochemicals.

Due to their limited solubility and stability, phytochemicals suffer from low bioavailability within the body. Therefore, new strategies of bioavailability improvement have been developed, such as delivery systems (nanoparticles, liposomes, and phytoliposomes) and co-administration with absorption enhancers. The development of dietary supplements in this field has garnered considerable interest. In line with this, a successful endeavor has been made to prepare a dietary capsule utilizing powdered extracts from berries and grape pomace. The resulting capsule incorporates a rich array of phenolic compounds, including chlorogenic acid (34.51%), rutin (19.13%), ferulic acid (17.03%), (+)-catechin (12.04%), and trace amounts (<10%) of other phenolic compounds. Moreover, this composition exhibits exceptional scavenging activity against the 2,2-diphenyl-1-picrylhydrazyl free radical (48.32 ± 0.74%). The particle size distribution and powder flow of the capsule meet the stringent requirements outlined by the European Pharmacopoeia, thus establishing a promising reference point for the industrial application of polyphenol-rich supplements [21].

The publications showcased in this Special Issue encompass a wide range of phytochemicals derived from various foods and plants. Collectively, these contributions significantly enhance our understanding of the bioactivities exerted by phytochemicals in the prevention of chronic diseases. However, further research efforts are required to fully harness their potential therapeutic efficacy. Advancing our comprehension of the benefits, mechanisms, and safety profiles associated with phytochemicals will undoubtedly make substantial advancements in human health and propel the development of the food, health food, and pharmaceutical industries.
